# Towards the prediction of essential genes by integration of network topology, cellular localization and biological process information

**DOI:** 10.1186/1471-2105-10-290

**Published:** 2009-09-16

**Authors:** Marcio L Acencio, Ney Lemke

**Affiliations:** 1 Department of Physics and Biophysics, São Paulo State University, Distrito de Rubiao Jr. s/n, Botucatu, São Paulo, Brazil

## Abstract

**Background:**

The identification of essential genes is important for the understanding of the minimal requirements for cellular life and for practical purposes, such as drug design. However, the experimental techniques for essential genes discovery are labor-intensive and time-consuming. Considering these experimental constraints, a computational approach capable of accurately predicting essential genes would be of great value. We therefore present here a machine learning-based computational approach relying on network topological features, cellular localization and biological process information for prediction of essential genes.

**Results:**

We constructed a decision tree-based meta-classifier and trained it on datasets with individual and grouped attributes-network topological features, cellular compartments and biological processes-to generate various predictors of essential genes. We showed that the predictors with better performances are those generated by datasets with integrated attributes. Using the predictor with all attributes, i.e., network topological features, cellular compartments and biological processes, we obtained the best predictor of essential genes that was then used to classify yeast genes with unknown essentiality status. Finally, we generated decision trees by training the J48 algorithm on datasets with all network topological features, cellular localization and biological process information to discover cellular rules for essentiality. We found that the number of protein physical interactions, the nuclear localization of proteins and the number of regulating transcription factors are the most important factors determining gene essentiality.

**Conclusion:**

We were able to demonstrate that network topological features, cellular localization and biological process information are reliable predictors of essential genes. Moreover, by constructing decision trees based on these data, we could discover cellular rules governing essentiality.

## Background

Essential genes are those genes required for growth in a rich medium, i.e., medium containing all nutrients required for growth. The deletion of only one of these genes is sufficient to confer a lethal phenotype on an organism regardless the presence of remaining genes. Therefore, the functions encoded by essential genes are crucial for survival and could be considered as a foundation of life itself [1,2]. The identification of essential genes is important not only for the understanding of the minimal requirements for cellular life, but also for practical purposes. For example, since most antibiotics target essential cellular processes, essential gene products of microbial cells are promising new targets for such drugs [3]. The prediction and discovery of essential genes have been performed by experimental procedures such as single gene knockouts [4], RNA interference [5] and conditional knockouts [6], but these techniques require a large investment of time and resources and they are not always feasible. Considering these experimental constraints, a computational approach capable of accurately predict essential genes would be of great value.

For prediction of essential genes, some investigators have implemented computational approaches in which most are based on sequence features of genes and proteins with or without homology comparison [7,8]. With the accumulation of data derived from experimental small-scale studies and high-throughput techniques, however, it is now possible to construct networks of gene and proteins interaction and then investigate whether the topological properties of these networks would be useful for predicting essential genes. Although many interaction networks have been built to date [9-12], most of studies relating essentiality with topological properties of these networks have been limited to indicate what topological properties are predictive of essentiality instead of using them as predictors of essential genes [9,13]. We have previously shown the feasibility of using network topological features for predicting essential genes in the bacterium *Escherichia coli *[14]. We have chosen *E. coli *as starting point for evaluating the prediction performance of essential genes by network topological features due to two reasons: the completeness of the catalog of *E. coli *essential genes [15] and the vast amount of interaction data available for this organism. In this present work, we sought to evaluate if network topological features can also be used as predictors of essential genes in the yeast *S. cerevisiae *since most of its genes have already been classified as essential or non-essential [4] and there are copious amounts of available interaction data for this organism.

For this purpose, we constructed a *S. cerevisiae *integrated network of gene interactions containing simultaneously protein physical, metabolic and transcriptional regulation interactions and used the topological features of this network as learning attributes in a machine learning-based prediction system. We tested individual and grouped network topological features as predictors of essential genes and showed that essential genes are best predicted by integrating the topological features in a single predictor. Although the prediction performance of topological features was shown to be acceptable, we added to this set of learning attributes data on cellular localization and biological process of genes in order to increase the predictability of essential genes. We found that the integration of network topology, cellular localization and biological process information in a single predictor increased the predictability of essential genes in comparison with the predictor containing only network topological features. Moreover, we observed that the predictability of essential genes by integration of cellular localization and biological process data in a single predictor was comparable to that of predictor containing network topological features.

Finally, in addition to study the predictability of essential genes, we tried to define some general rules governing essentiality in *S. cerevisiae *by analyzing decision trees generated by a machine learning-based technique. Using network topology, cellular localization and biological process information as training attributes, we discovered that essentiality depends on the number of protein physical interactions, the nuclear localization of proteins and the number of regulating transcription factors. Taken together, all these findings show that the integration of network analysis along with cellular localization and biological process information is a powerful tool for both predicting biological characteristics of genes, such as essentiality, and discovering the biological determinants of phenotypes.

## Results and Discussion

### Integrated network of gene interactions in S. cerevisiae and calculation of topological features

For obtaining the network topological features used as training data for predicting essential genes, we first constructed an integrated network of gene interactions (INGI) of *Saccharomyces cerevisiae *simultaneously containing experimentally verified protein physical interactions, metabolic interactions and transcriptional regulation interactions (definitions for each type of interaction are detailed in "Methods"). This network is comprised by 5,667 genes interacting with one another via 42,893 protein physical interactions, 11,192 metabolic interactions and 18,721 transcriptional regulation interactions. Of 5,667 genes in the network, 5,637 (99,5%) are protein-coding genes (including transposable elements), 15 (0.26%) are transfer RNA-coding genes, 13 (0.23%) are small nucleolar RNA-coding genes and 2 (0.01%) are RNA-coding genes of unknown function. Regarding protein-coding genes, including transposable elements, our network contains 96% of the total 5,884 protein-coding genes of *S. cerevisiae *according to the current status of the yeast genome provided by the *Saccharomyces *Genome Database (SGD) [16].

We calculated 12 different topological features for each gene in the INGI, including degree centralities for each type of interaction, clustering coefficient, betweenness centralities for each type of interaction, closeness centrality and identicalness. The detailed description of these topological features and how they were calculated are found in the Additional file 1 and "Methods".

### Comparison of the classification performance among balanced datasets

**Figure 1:**
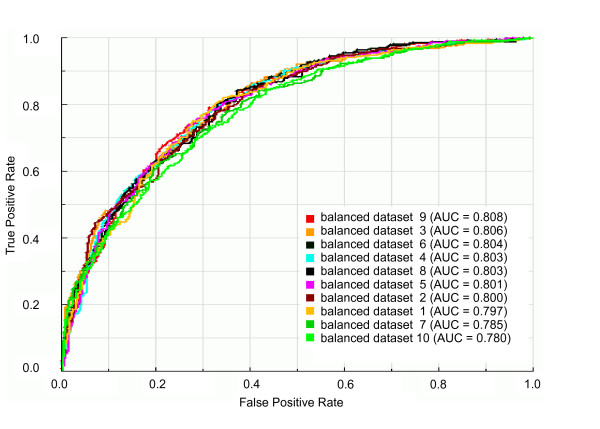
**ROC curves and AUC values for classifiers trained on the ten balanced datasets with all available learning attributes**. Balanced datasets 1-10: datasets with all available learning attributes prepared by an undersampling scheme as described in "Methods".

The performance of machine learning-based approaches is known to be affected by imbalanced data [17]. As the dataset containing yeast genes classified into essential and non-essential genes intended to be used as training data for our classifier is an imbalanced dataset, we used an undersampling scheme to generate ten balanced datasets from the original data (see "Methods"). Each balanced dataset contains different subsets of non-essential genes as a result of the sampling approach. Due to these different subsets of non-essential genes, therefore, we statistically compared the prediction performance of balanced datasets before assessing the predictability of essential genes by the different features. We trained our classifier on each of the balanced dataset with all available training data (network topological features and cellular localization and biological process information) and evaluated the prediction performance of each balanced dataset. Comparing the Area Under the receiver operating characteristic (ROC) Curve (AUC) values among all the balanced datasets (Figure 1 and Additional file 2), we verified that their prediction performances are not statistically different as evaluated by a nonparametric statistical method based on the Mann-Whitney U-statistic [18] (see more details in "Methods"). Based on these results, we selected one of the balanced datasets to perform the following analyses.

### Prediction of essential genes by network topological features

**Figure 2:**
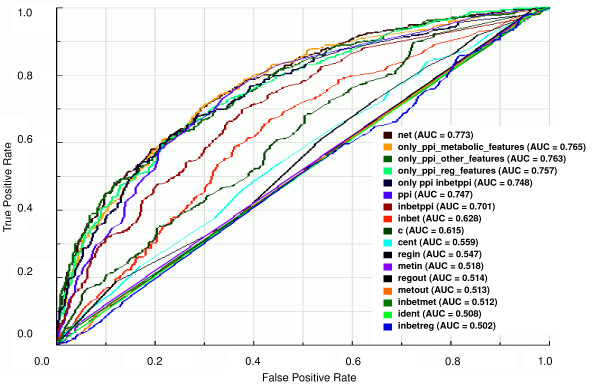
**ROC curves and AUC values for the classifiers trained on balanced datasets with individual or grouped network topological features**. ROC curves and AUC values of classifiers trained on balanced dataset 9 (see Figure 1) with one or groups of network topological features as learning attributes as follows: "net": all network topological features as learning attributes; "ppi", "inbetppi", "inbet", "c", "cent", "regin", "metin", "regout". "metout", "inbetmet", "ident" and "inbetreg": datasets with only one of the following network topological features as learning attribute: number of protein physical interactions (*ppi*), betweenness centrality for the protein physical interactions (*inbetppi*), betweenness centrality for all types of interactions (*inbet*), clustering coefficient (*c*), closeness centrality (*cent*, number of regulating transcription factor (*regin*), number of reactants participating in a metabolic reaction catalyzed by the enzyme encoded by the gene (*metin*), number of genes regulated by the transcription factor encoded by the gene (*regout*), number of products generated in a metabolic reaction catalyzed by the enzyme encoded by the gene (*metout*), betweenness centrality for the metabolic interactions (*inbetmet*), number of genes with identical topological features (*ident*) and betweenness centrality for the transcriptional regulation interactions (*inbetreg*). "only _ppi_metabolic_features" and "only_ppi_reg_features": datasets containing protein physical interactions-related features (*ppi *and *inbetppi*) and, respectively, metabolic (*met*, *metin*, *metout *and *inbetmet*) and transcriptional regulatory interactions-related features (*reg*, *regin*, *regout *and *inbetreg*). "only_ppi_other_features": dataset containing protein physical interactions-related features (*ppi *and *inbetppi*) and *c*, *ident*, *cent *and *inbet*. "only_ppi_inbetppi": dataset containing only the indicated network topological features as learning attributes. For more details on network topological features, see Additional file 1.

We started the analyzes by assessing the predictability of essential genes by each of the 12 network topological features (computed as described in " Methods") and by all 12 network topological features integrated in a single predictor. For this purpose, we trained our classifier on a balanced dataset with all network topological features as training data and on a dataset containing only one of the network topological features as training data (see "Methods" for detailed information on construction of the balanced datasets). The ROC plot shown in Figure 2 indicates that integration of all networks topological features in a single predictor outperforms the predictability of essential genes by the individual network topological features. By comparing the AUC values of grouped and individual network topological features, we verified that the AUC value of grouped network topological features (AUC = 0.773) is statistically significantly higher (*P *< 0.002) than AUC value of any individual network topological feature (Figure 2 and Additional file 2).

We then verified if different combinations of grouped network topological features could show prediction performances comparable to that of all grouped network topological features. We found that the combination of protein physical interactions-related features with metabolic interactions-related features has the same performance (AUC = 0.765, *P *= 0.302; see Additional file 2 and Figure 2) seen for the predictor containing all grouped network topological features (AUC = 0.773). Also, the combination of protein physical interactions-related features with clustering coefficient, identicalness and betweenness and closeness centralities has the same prediction performance (AUC = 0.763, *P *= 0.071; see Additional file 2 and Figure 2) observed for all grouped network topological features (AUC = 0.773). Therefore, smaller sets of network topological features can be used to predict essential genes, thus making the calculation of all topological features dispensable.

To verify if the predictive power of all grouped network topological features could be improved by exclusion of topological features with marginal AUC values, i.e., AUC values ranging from 0.500 to 0.600, we compared the prediction performance of all grouped network topological features (AUC = 0.773) with those of the combinations of features in which one feature or a small set of features was excluded (see the correspondent ROC curves in the Additional file 3 and the pairwise comparison of predictors with the p-values of AUC differences between each pair of predictors in Additional file 2). We discovered that the prediction performance of all grouped network topological features is not improved by the removal of any individual or small sets of topological features (see Additional files 2 and 3). As expected, the exclusion of grouped features related to metabolic interactions or grouped features related to protein physical interactions diminishes (AUC = 0.764; *P *= 0.002 and for metabolic interaction-related features and AUC = 0.749; *P *= 0.001 for protein physical interaction-related features) the prediction performance of all grouped network topological features (AUC = 0.773).

Among all individual network topological features, the number of protein physical interactions is that one that best predicts essential genes (AUC = 0.747). As further discussed in "Cellular rules for essentiality", other investigators have shown that the number of physical interactions is indicative of essentiality [9,19,20]. To our knowledge, we are the first to compare the number of protein physical interactions with other network topological features. Despite the good performance of number of protein physical interactions on predicting essential genes among other individual network topological features, the best predictors are those integrating other groups of topological features with the number of protein physical interactions. This indicates that essentiality depends more or less on each network topological feature and, therefore, the gene location in the network seems to be important for determining its essentiality.

### Prediction of essential genes by cellular localization and biological process data

Although the prediction performance of the integrated network topological features in a single predictor can be considered acceptable for predicting essential genes, we decided to check if the addition of information on cellular localization and biological process as training data would increase the predictability of essential genes. Before integrating cellular localization and biological process data with network topological data, we assessed the individual performance of each cellular component and each biological process, as well as the collective performance of all cellular components and all biological processes on predicting essential genes, in order to verify if any individual feature or grouped features related to cellular localization or biological process are good predictors of essential genes.

**Figure 3:**
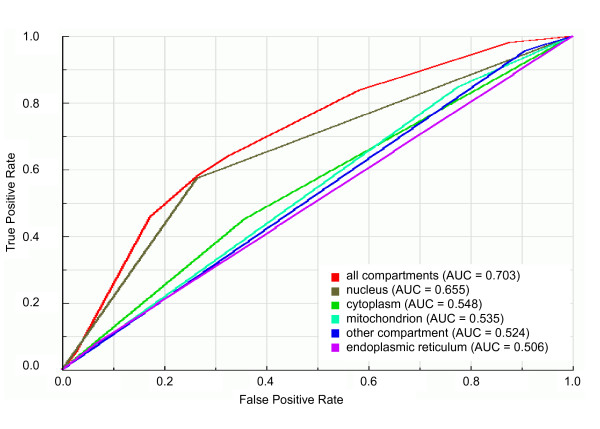
**ROC curves and AUC values for the classifiers trained on balanced datasets with individual or grouped cellular compartments**. ROC curves and AUC values of classifiers trained on balanced dataset 9 (see Figure 1) with one or all cellular compartments as learning attributes. "all compartments" is the dataset with all cellular compartments as learning attributes; "nucleus", "cytoplasm", "mitochondrion", "other compartment" and "endoplasmic reticulum" are datasets with only the respective cellular compartment as learning attribute.

**Figure 4:**
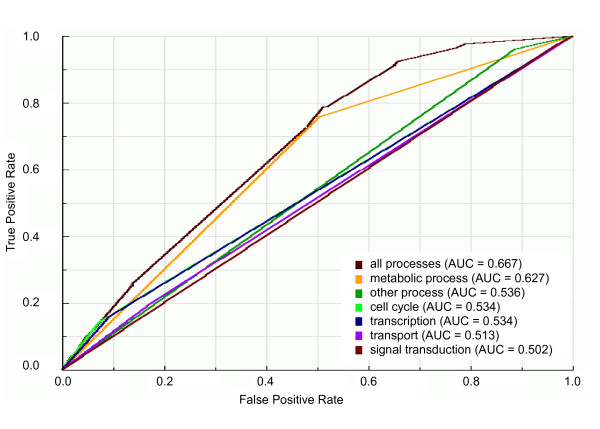
**ROC curves and AUC values for the classifiers trained on balanced datasets with individual or grouped biological processes**. ROC curves and AUC values of classifiers trained on balanced dataset 9 (see Figure 1) with one or all biological processes as learning attributes. "all processes" is the dataset with all biological processes as learning attributes; "metabolic process", "other process", "cell cycle", "transcription" and "transport" are datasets with only the respective biological process as learning attribute.

Regarding cellular localization, we trained our classifier on balanced datasets with all cellular compartments as training data (cytoplasm, endoplasmic reticulum, mitochondrion, nucleus or other localization) and on datasets containing only one of the cellular compartments as training data. We can observe in the ROC plot shown in Figure 3 that the best predictor of essential genes seems to be the integrated set of cellular compartments. This is confirmed by the statistical comparison of the AUC value of the integrated set of cellular compartments with those of individual cellular compartments: the AUC value of grouped cellular compartments (AUC = 0.703) is significantly (*P *< 10^-5^) higher than AUC values of any individual cellular compartment (Figure 3 and Additional file 2), although such AUC value characterizes the set of all cellular components as fair predictors of essential gene prediction. With regard to biological processes, we trained our classifier on balanced datasets with all biological processes as training data (cell cycle, metabolic process, signal transduction, transcription, transport or other process) and on datasets containing only one of the biological processes as training data. The ROC curves for biological processes (Figure 4) show the same behavior observed for the prediction of essential genes by both network topological features and cellular compartment: the integration of attributes in a single predictor increases the predictability of essential genes in comparison with predictability by individual attributes. The AUC value of the integrated set of biological processes (AUC = 0.667) is statistically significantly (*P *< 0.001) higher than AUC values of any individual biological process (Figure 4 and Additional file 2). With the AUC value of 0.667, however, the set of biological processes can be considered a poor predictor of essential genes.

**Figure 5:**
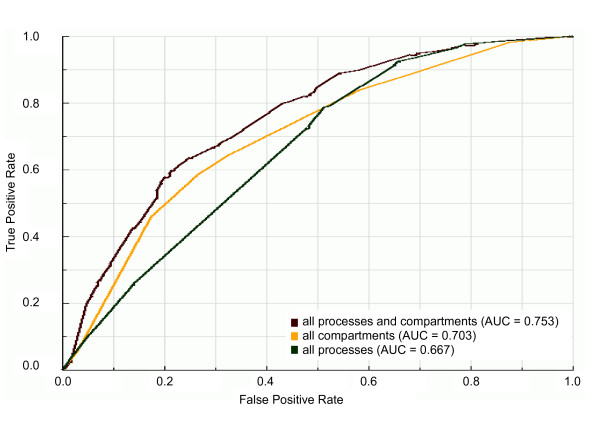
**ROC curves and AUC values for the integrated predictors with cellular localization and biological process information**. ROC curves and AUC values of classifiers trained on balanced dataset 9 (see Figure 1) with all biological processes ("all processes"), all cellular compartments ("all compartments") or all biological processes and cellular compartments ("all processes and compartments") as learning attributes.

The moderate and poor performances of cellular localization and biological processes as predictors of essential genes, respectively, suggest that essentiality, as further discussed in "Cellular rules for gene essentiality", is probably a result of multiple factors, reinforcing what we found by analyzing the prediction performance of network topological features. Therefore, we decided to evaluate the prediction performance of the integration of cellular localization and biological process information in a single predictor. We then trained our classifier on balanced datasets with all cellular compartments and biological processes as training data. Figure 5 indicates that the performance of integration of cellular localization and biological process data on predicting essential genes is better than other predictors. In fact, the AUC value of predictor containing all cellular localization and biological processes data (AUC = 0.753) is statistically higher (*P *< 10^-5^) than AUC values of other predictors (see Additional file 2).

### Prediction of essential genes by integrating network topological features, cellular localization and biological process information

After determining the predictive power of individual and grouped cellular localization and biological process data, we sought to verify if integration of network topological features with cellular localization and biological process data in a single predictor would improve predictability of essential genes. Moreover, we also sought to compare the predictability of essential genes by all network topological features integrated in a single predictor with that by all cellular compartments and all biological processes integrated in a single predictor. It is worth to mention that although we choose the predictor containing all network topological features to perform the following comparisons, the sets containing protein physical interactions-related features with metabolic interactions-related features or other features (see "Prediction of essential genes by network topological features" for details) also could be used since their prediction performances are comparable to that of all grouped network topological features.

**Figure 6:**
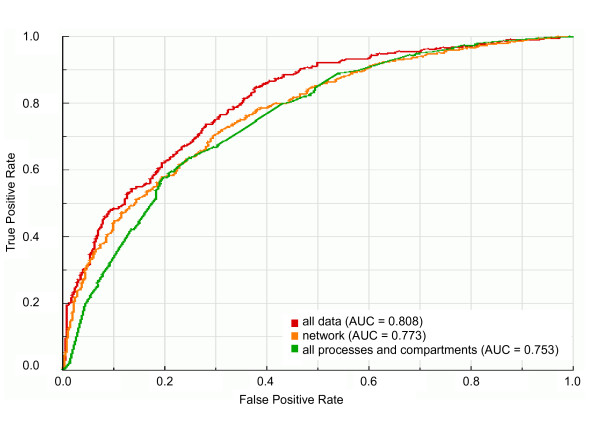
**ROC curves and AUC values for the integrated predictors with available data**. ROC curves and AUC values of classifiers trained on balanced dataset 9 (see Figure 1) with all network topological features, cellular compartments and biological processes ("all data"), all biological processes and cellular compartments ("all processes and compartments") or all network topological features ("network") as learning attributes.

For evaluating the integration of all data in a single predictor and comparing it with the predictor containing only cellular localization and biological process information and with the predictor containing only network topological features, we trained our classifier on balanced datasets with all available data as training data, all cellular compartments and biological processes as training data and all network topological features, cellular components and biological processes as training data. As expected, the ROC curves in Figure 6 indicate that integration of all network topological features with cellular compartments and biological processes information in a single predictor increases the predictability of essential genes in comparison with predictors containing only network topological features or cellular compartments and biological processes information. Indeed, comparing the AUC value of predictor containing all network topological features and all cellular compartments and biological processes information with that of predictor containing only network topological features or cellular compartments and biological processes information, we confirmed that predictability of essential genes by the integrated predictor (AUC = 0.808) is statistically significantly (*P *< 10^-4^) higher than that by others predictors (Figure 6 and Additional file 2).

Regarding the comparison of the predictive power of integrated topological network features with that of integrated cellular localization and biological process data, we observed that the difference between the AUC value of predictor containing all cellular compartments and biological processes information (AUC = 0.753) and the AUC value of predictor containing all network topological features (AUC = 0.773) is not statistically significant (*P *= 0.269) (see Additional file 2). Considering that the function of a protein is intimately linked to its cellular localization [21] and that both the biological process in which a protein is involved and the cellular localization in which a protein acts are predictable by network topological features [10,22], it is not surprising that the predictabilities of essential genes by both the predictor containing all network topological features and the predictor containing all cellular localization and biological process data are similar.

### Classification of yeast genes not known to be essential

We obtained the list of genes classified as essential and non-essential used for training our classifier from Giaever *et al*. [4] (see "Methods"). Giaever *et al*. have systematically constructed a nearly complete collection of yeast gene-deletion mutants covering about 96% of all genes. However, about 430 genes of this collection were removed from the yeast genome after a comprehensive reannotation process of the *S. cerevisiae *genome performed in 2006 [23]. In addition, new genes were annotated to yeast genome as a result of this reannotation process. In order to classify these genes not analyzed by Giaever *et al*., we used our best classifier, that is, the one that containing all network topological features, cellular components and biological processes information as training attributes. For each gene, the predictor output the probability of classifying it as essential and non-essential, which we called, respectively, "essentiality score" and "non-essentiality score".

To predict a gene as essential, we defined an essentiality score of 0.654 as the cutoff value, i.e., genes with essentiality score above 0.654 were considered to be essential. This cutoff value was based on the optimal threshold, which is the score value that leads to the maximal accuracy of classification, calculated by the software StAR [24] for the predictor containing all features (network topological, cellular component and biological process; see Figure 6 and Additional file 2). Among the 514 genes with the essentiality status not defined by Giaever *et al*., 44 genes were predicted as essential (Table 1). Analyzing these genes, we found that 9 genes have been previously demonstrated to be essential (*YHR165C*, *YHR089C*, *YHR052W*, *YCR042C*, *YDR320C-A*, *YHR169W*, *YKL138C-A*, *YGL106W *and *YHR099W*) and other 14 genes (*YGR252W*, *YHR027C*, *YOL012C*, *YNL147W*, *YGL100W*, *YNL096C*, *YOL148C*, *YFL007W*, *YOL145C*, *YBR111W-A*, *YNL055C*, *YHR216W*, *YBL071W-A *and *YHR039C-A*) have been previously demonstrated to be non-essential by other investigators through small-scale gene deletion experiments in functional characterization studies [25-36] (Table 1). Among non-essential genes, 10 genes (*YGR252W*, *YHR027C*, *YOL012C*, *YNL147W*, *YNL096C*, *YOL148C*, *YOL145C*, *YBR111W-A*, *YNL055C *and *YHR039C-A*) have been shown to impair substantially the growth of *S. cerevisiae *when they are completely deleted [33,36-40], whereas the 4 remaining non-essential genes (*YGL100W*, *YFL007W*, *YHR216W *and *YBL071W-A*) have been shown not to affect the growth phenotype of yeast when they are deleted [34,35,41,42]. Although roughly 1/3 of the these genes predicted to be essential have been previously classified as non-essential, the complete deletions of most of them have been shown to severely reduce the fitness of organisms [33,36-40], suggesting that our predictor, even when directly contradicted by these experimental findings, can nonetheless identify genes important to cellular function. Regarding the 4 non-essential genes whose deletion has been shown not to affect the growth phenotype of yeast (*YFL007W *and *YGL100W*), we hypothesize that our classifier assigned a high essentiality score to these genes due to the following features: (*i*) their encoded proteins interact with more than 12 other proteins, (*ii*) they are regulated by less than 4 transcription factors and (*iii*) their encoded proteins are located in the nucleus. These characteristics are in accordance with two cellular rules for essentiality discovered by our approach as demonstrated in the section "Cellular rules for gene essentiality": if proteins interact with more than 7 other proteins and are located in the nucleus, genes encoding them are likely to be essential and genes regulated by more than 3 transcription factors tend to be non-essential.

**Table 1:** List of the 44 yeast genes predicted to be essential in S. cerevisiae

Rank	Gene	Essentiality Score	Essentiality Status	Deletion phenotype	Reference
1	YHR165C	0.940	essential	lethality	[32]
2	YGR252W	0.939	non-essential	defective growth	[33]
3	YHR089C	0.937	essential	lethality	[25]
4	YHR052W	0.065	essential	lethality	[26]
5	YER029C	0.930	not defined	not defined	-
6	YHR027C	0.930	non-essential	defective growth	[37]
7	YHR099W	0.929	essential	lethality	[27]
8	YOL012C	0.925	non-essential	defective growth	[33]
9	YHR169W	0.921	essential	lethality	[28]
10	YCR042C	0.920	essential	lethality	[29]
11	YDR320C-A	0.897	essential	lethality	[30]
12	YNL147W	0.885	non-essential	defective growth	[33]
13	YGL100W	0.866	non-essential	not related to growth	[41]
14	YNL096C	0.865	non-essential	defective growth	[33]
15	YOL148C	0.859	non-essential	defective growth	[38]
16	YOR145C	0.856	essential	lethality	[31]
17	YFL007W	0.839	non-essential	not related to growth	[42]
18	YKL138C-A	0.837	essential	lethality	[30]
19	YOL145C	0.824	non-essential	defective growth	[39]
20	YBR111W-A	0.822	non-essential	defective growth	[40]
21	YLL022C	0.816	not defined	not defined	-
22	YNL209W	0.816	not defined	not defined	-
23	YGL106W	0.813	not defined	not defined	-
24	YPR080W	0.813	not defined	not defined	-
25	YER105C	0.794	not defined	not defined	
26	YNL055C	0.783	non-essential	defective growth	[33]
27	YOL142W	0.781	not defined	not defined	-
28	YAL024C	0.770	not defined	not defined	
29	YHR216W	0.768	non-essential	defective growth	[34]
30	YHL004W	0.743	not defined	not defined	-
31	YHR072W-A	0.741	not defined	not defined	-
32	YGL190C	0.738	not defined	not defined	-
33	YDR079C-A	0.731	not defined	not defined	-
34	YNL186W	0.731	not defined	not defined	-
35	YJR132W	0.716	not defined	not defined	-
36	YDR261W-A	0.713	non-essential	defective growth	[33]
37	YHR119W	0.696	not defined	not defined	-
38	YBL071W-A	0.693	non-essential	defective growth	[35]
39	YDR261W-B	0.682	non-essential	defective growth	[34]
40	YHR039C-A	0.680	non-essential	defective growth	[36]
41	YHR090C	0.680	not defined	not defined	-
42	YER026C	0.675	not defined	not defined	-
43	YHR056C	0.665	not defined	not defined	-
44	YCL019W	0.659	not defined	not defined	-

Among the 44 genes predicted to be essential, 21 genes have not yet been investigated for essentiality to date (Table 1). One of these genes is the *YER029C *whose encoded protein (Yer029cp) binds to other 6 proteins to form the heteroheptameric complex that is required for the biogenesis of the spliceosomal U1, U2, U4, and U5 snRNPs [43]. These spliceosomal snRNPs are involved in splicing of nuclear pre-mRNAs [44], an essential biological process for cell viability, and, interestingly, all proteins forming the heteroheptameric complex along with Yer029cp have been demonstrated to be essential [4]. Therefore, the presence of this gene among ones predicted to be essential reinforces the fact that our predictor is able to identify genes that are important to cellular function.

Finally, regarding the remaining 470 genes predicted as non-essential, we verified that 129 of these genes have been previously tested for essentiality by other studies (see Additional file 4). Among them, 124 have been demonstrated to be non-essential genes and only 5 have been demonstrated to be essential genes. Thus, about 4% of genes with known essentiality status and predicted as non-essential are actually essential genes (Additional file 4). Providing that 38% (9 of 14; see Table 2) of the genes with known essentiality status and predicted as essential are actually essential genes, the predictor integrating all available features (network topological, cellular component and biological process; see Figure 6 and Additional file 2) leads to an enrichment of actual essential genes in the set of genes predicted as essential. This suggests that this predictor is committed to minimize the false negative rate thus avoiding the loss of essential genes.

### Cellular rules for gene essentiality

Beyond the prediction capability, machine learning techniques can be used for knowledge acquisition in order to describe patterns in datasets. The machine learning algorithms most used for knowledge acquisition are those that generate decision trees. Decision trees are decision support tools inferred from the training data that use a graph of conditions and their possible consequences. The structure of a decision tree consists of a root node representing the most important condition for discriminating classes, internal nodes representing additional conditions for class discrimination under the main condition, and leaf nodes representing the final classification. So, one can learn the conditions for classifying instances in a given class by following the path from the root node to the leaf node [45].

Therefore, in order to discover the rules for gene essentiality in *S. cerevisiae*, we analyzed decision trees generated by training the J48 algorithm, a WEKA's implementation of the C4.5 algorithm [46] (for more details, see "Methods"), on the ten balanced datasets containing all network topological features, cellular components and biological processes as training data (the construction of balanced datasets are detailed in "Methods"). As decision trees generated from the balanced datasets could be slightly different from one another due to the undersampling scheme used to balance the original set of classified genes--each balanced dataset contains a different set of 1,024 non-essential genes, 1/8 of the total amount in the original imbalanced dataset--we generated one detailed (64 instances per leaf) and one simplified (128 instances per leaf) decision tree for each balanced dataset (see "Methods" for details) and then we manually inspected them in order to discover the general rules for gene essentiality.

**Figure 7:**
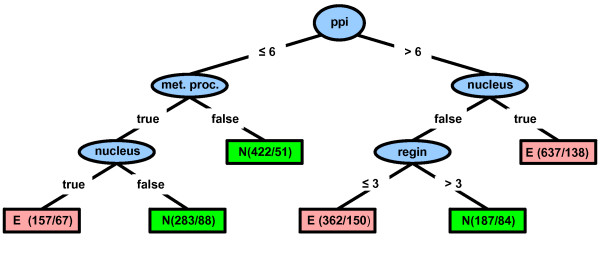
**Decision tree generated by training the J48 algorithm on the balanced dataset 8 with all available data**. This decision tree was generated by training the J48 algorithm on the balanced dataset 8 with all available data (see "Methods"). The uppermost ellipse is the node root of tree that represents the most important condition for discriminating essential genes from non-essential genes. In this case, such condition is the number of protein physical interactions (*ppi*). The remaining ellipses are internal nodes that represent additional conditions for considering a gene as essential or non-essential. In the left branch of tree, such conditions are involvement in a metabolic process (*met. proc*.) and nuclear localization (*nucleus*). In the right branch, such conditions are nuclear localization (*nucleus*) and number of regulating transcription factors (*regin*). The rectangles are the leaf nodes that represent the final classification. Red and green rectangles depict genes that, under certain conditions (represented by the root node and internal nodes), are respectively and predominantly classified as essential (**E**) and non-essential (**N**). In the round brackets inside rectangles, the number before the slash indicates the total number of genes that are actually essential or non-essential and the number after the slash indicates how many genes were incorrectly predicted.

From the 20 slightly different generated decision trees, we were able to devise the general rules for gene essentiality in *S. cerevisiae*. Figure 7 shows the decision tree that best illustrates the general rules for gene essentiality (all decision trees are available in text format in the Additional file 5). As we can observe in Figure 7, the root node of decision tree is the number of protein physical interactions (all generated decision trees exhibit this feature; see Additional file 5); so, this attribute can be considered the most important feature among all network topological features and cellular localization and biological process information for gene essentiality. Accordingly, the predictor containing only the number of protein physical interaction as training feature is the one that best predicts (AUC = 0.747) essential genes among all other individual features as we can observe in Figure 2. This is in concert with previous studies that have demonstrated that the number of protein physical interactions is indicative of essentiality [9,19,20]. Several hypotheses about the connection between gene essentiality and number of protein physical interactions have been proposed. Coulomb *et al*. [47] have suggested that the relationship between this network feature and gene essentiality is partly due to biases in the interaction data that are enriched in small-scale experiments which are partial towards essential genes. On the other hand, Zotenko *et al*. [48] have recently hypothesized that the connection between gene essentiality and number of protein physical interactions is likely due to the involvement of proteins encoded by essential genes in subnetworks of densely connected proteins with shared biological functions that are enriched in proteins encoded by essential genes.

Following the path from root node to first leaf node through the right branch (Figure 7), we found the following rule for gene essentiality: if proteins interact with more than 7 other proteins (average of number of interactions ranging from 6 to 12 in all decision trees) and are located in the nucleus, genes encoding them are likely to be essential. This rule can be observed in 9 of 10 decision trees with 128 instances per leaf and 8 of 10 decision trees with 64 instances per leaf (see Additional file 5). If these proteins are located in cellular compartments other than the nucleus, essentiality of their corresponding genes depends on conditions particular to each decision tree (Figure 7 and Additional file 5). The path from root node to the leaf nodes through the left branch (Figure 7) drove us to discover another rule for gene essentiality: if proteins interact with 6 or fewer proteins and participate in a metabolic process inside the nucleus, genes encoding these proteins are likely to be essential. This rule can be observed in 7 of 10 decision trees with both 128 and 64 instances per leaf (Additional file 5).

According to these rules, the ultimate condition for gene essentiality is the localization of proteins in the nucleus, suggesting that this cellular component is somehow important for essentiality. The importance of nucleus for essentiality has also been suggested by Seringhaus *et al*. [7] that have shown that nuclear localization has the strongest positive correlation with essentiality among other cellular components. The relationship between nucleus and essentiality can be explained by the fact that roughly one third of nuclear proteins are encoded by essential genes and most of essential biological processes for cell viability take place within the nucleus [49]. Therefore, the participation of proteins in these nuclear-localized essential processes, such as DNA replication, transcription and DNA repair, should be a pivotal condition for essentiality in the rules defined by both the paths via the left and right branches of decision tree. It is worth to mention that, as a result of the annotation method we used (see more details in "Methods"), these nuclear-localized essential processes are embedded in the biological process "metabolic process", one of the conditions for essentiality along with nuclear localization and number of protein physical interactions equal or less than 6 in the rule defined by the path via the left branch of decision tree (Figure 7). In the rule defined by the path via the right branch, although essentiality is apparently not dependent on the involvement of proteins in metabolic processes inside the nucleus, the nuclear proteins encoded by genes classified as essential according to this rule may be actually involved in a nuclear metabolic process. In this case, however, the involvement in nuclear metabolic processes is overwhelmed by the number of protein physical interactions.

We discovered an additional interesting rule for gene essentiality in yeast: genes regulated by more than 3 transcription factors tend to be non-essential (Figure 7). This rule can be observed in 6 of 10 decision trees with 128 instances per leaf and in all decision trees when the number of instances per leaf is set to 64 (see "Methods" for details and Additional file 5). Our finding is corroborated by Yu *et al*. [50] that have found that genes regulated by > 10 transcription factors are less likely to be essential than those regulated by 2-9 transcription factors, whereas these genes are less likely to be essential than those with only one transcription factor. At first glimpse, the fact that essential genes tend to be regulated by a few transcription factors seems contradictory since one would expect that gene essentiality is correlated with a high level of transcriptional regulation. However, most essential genes encode housekeeping proteins, i.e., proteins involved in housekeeping functions, such as rRNA metabolic process and transcription initiation [48]. As housekeeping functions are the most basic and important functions within cell, genes encoding housekeeping proteins are ubiquitously expressed and, consequently, they tend to be regulated by fewer transcription factors than genes encoding non-housekeeping proteins. Therefore, this phenomenon is likely due to the enrichment of genes encoding housekeeping proteins in the set of essential genes.

## Conclusion

The identification of essential genes has largely been an experimental effort mostly performed by time-consuming whole-genome knockout experiments. In an effort to accelerate the pace of discovery of essential genes, we designed a machine learning-based computational approach that relies on network topological features, cellular localization and biological process information for predicting essential genes and evaluated it in the yeast *Saccharomyces cerevisiae*.

We therefore constructed an integrated network of gene interactions for *S. cerevisiae *containing protein physical, metabolic and transcriptional regulation interactions and computed 12 different network topological features (as described in Additional file 1 and "Methods") that were individually and collectively evaluated for their ability to predict essential genes. We showed that the predictors containing all 12 network topological features or different combinations of protein physical interactions-related features with other groups of topological features as training data are reliable predictors (AUC = 0.763-0.773) of essential genes in *S. cerevisiae*, thus reinforcing the fact that an integrated network of gene interactions can be an useful tool for the prediction of essential genes.

Although the performance of predictors containing only network topological features can be considered acceptable for predicting essential genes, we decided to check if the addition of cellular localization and biological process information to these predictors would increase the predictability of essential genes. In fact, we verified that the performance of the predictor containing all network topological features, cellular localization and biological process information as training data is better than those of the predictors containing only network topological features or only cellular localization and biological process information. Interestingly, we also showed that the prediction performances of the predictor containing only network topological predictions and the predictor containing only cellular localization and biological process information are similar. To our knowledge, this is the first time that Gene Ontology terms related to cellular localization and biological process are shown to be useful predictors of essential genes.

In addition to prediction of essential genes, we could also devise some cellular rules for gene essentiality using all network topological features, cellular localization and biological process information as training data for generation of decision trees (see details in section "Cellular rules for gene essentiality"). We discovered that the number of protein physical interactions, the nuclear localization and the number of regulating transcription factors are important factors determining gene essentiality. Although these findings have previously been demonstrated by other investigators [7,9,19,20,50], it is interesting to notice that we were able to obtain these same results by simply inspecting the decision tree generated as shown in section "Cellular rules for gene essentiality". So, decision trees are useful tools for extracting knowledge from complex biological data.

Besides confirming previous findings, the exploration of decision trees can also lead to new discoveries. This can be exemplified by an additional analysis that we performed due to a referee' s suggestion regarding the nuclear localization of essential proteins. The referee has suggested us to analyze the influence of some children terms of GO term "nucleus" on the nuclear localization-related gene essentiality. For this purpose, we generated a decision tree by training the J48 algorithm on one of the ten balanced datasets (see "Methods" for details) with all features plus the GO terms "nucleolus", "nucleoplasm", "nuclear chromosome" and "nuclear envelope" and, as can be observed in the Additional file 5, an entirely new rule can be devised from the generated decision tree: the nucleolar localization of proteins is the most important factor for gene essentiality. We did not mention this potential and interesting rule for gene essentiality in the section "Cellular rules for gene essentiality" since this rule *per se *is interesting enough to deserve a more exhaustive analysis that can be reported in a future paper.

Albeit the good prediction performance and the ability to discover cellular rules for essentiality, our approach suffers from two limitations. First, it depends on existing Gene Ontology annotation and protein physical interaction data which are likely to be enriched in small-scale experiments involving essential genes. Second, the construction of an integrated network of gene interactions requires a large amount of experimental interaction data that are currently available only to a limited number of organisms.

Therefore, the prediction of essential genes in newly sequenced organisms, for example, is impractical by our approach. However, the integration of our approach with (*i*) computational-based methods for gene annotation and (*ii*) computational-based methods for the construction of integrated networks of predicted gene interactions in which each type of interaction (protein physical, metabolic and transcriptional regulation interactions) can be distinguished from one another could give rise to a purely *in silico *network topology, cellular localization and biological process information-based methodology for prediction of essential genes. Such a methodology would be totally independent on experimental interaction data and, accordingly, unbiased in essential genes-driven experiments.

In summary, despite the limitations discussed above, we could demonstrate that the integration of network topological features, cellular localization and biological process information is capable to predict essential genes. In this work, we tested the predictive performance of this integration in *S. cerevisiae*, but we envisage that it might be useful to predict essential genes in any other organism if a purely computational-based prediction approach, as suggested above, is used.

## Methods

### Generation of the set of training features

#### Network topological features

In order to compute the network topological features used as training features for predicting essential genes, we first constructed an integrated network of gene interactions of *S. cerevisiae *based on assumption that two genes, *g*_1 _and *g*_2_, coding respectively for proteins *p*_1 _and *p*_2_, are interacting genes if *(i) p*_1 _and *p*_2 _interact physically (protein physical interaction), (*ii*) the transcription factor *p*_1 _directly regulates the transcription of gene *g*_2_, i.e., *p*_1 _binds to the promoter region of *g*_2 _(transcriptional regulation interaction), or (*iii*) the enzymes *p*_1 _and *p*_2 _share metabolites, i.e., a product generated by a reaction catalyzed by enzyme *p*_1 _is used as reactant by a reaction catalyzed by enzyme *p*_2 _(metabolic interaction).

Yeast protein physical interactions data were obtained from The Biological General Repository for Interaction Datasets (BioGRID) database, a repository of literature-curated protein physical and genetic interactions [51]. We downloaded the database release 2.0.42 of July 2008 and removed the entries related to genetic interactions. Yeast transcriptional regulation interactions were obtained from the Yeast Search for Transcriptional Regulators And Consensus Tracking (YEASTRACT) database, a curated repository of regulatory associations between transcription factors and target genes in *Saccharomyces cerevisiae *[52]. By using the utility "Generate Matrix Regulation" in the YEASTRACT website, we generated and downloaded a regulation matrix containing only documented transcriptional regulation interactions determined by direct experimental evidence.

Yeast metabolic interactions were extracted from the metabolic model iND750 of *Saccharomyces cerevisiae *[11] by a code implemented in Mathematica^® ^6.0 (Wolfram Research, Inc.). We excluded those metabolic interactions generated by the so-called "currency metabolites", abundant molecular species present throughout the cell most of the time and, therefore, unlikely to impose any constraints on the dynamics of metabolic reactions. Due to this feature of currency metabolites, the functionality of the network would be better represented without them [53]. We considered currency metabolites the eight most connected metabolites (ADP, ATP, H^+^, H_2_O, NADP^+^, NADPH, orthophosphate and pyrophosphate) in the original metabolic model iND750.

The final integrated network of gene interactions (INGI) of yeast is the result of integration of the protein physical, metabolic and transcriptional regulation interactions datasets through genes common to these datasets. Before performing the integration, we converted all yeast gene names to their systematic names--as provided by the Saccharomyces Genome Database (SGD) Nomenclature Conventions [23]--to avoid the creation of false interactions due to gene name ambiguity. Genes classified as dubious, i.e., genes unlikely to encode an expressed protein and not considered biologically significant by SGD, were removed from the final INGI.

For each gene *g *in the yeast INGI, we computed twelve network topological features as listed in Additional file 1. Briefly, degree centrality is defined as the number of links to node (in our case, gene). We considered each type of interaction as a distinct measure of degree as described in Additional file 1. Clustering coefficient (*c*) of a node (in our case, a gene) quantifies how close the node and its neighbors are to being a clique, i.e., all nodes connected to all nodes. For yeast INGI, *c *is defined as the proportion of links between the genes within the neighborhood of *g *divided by the number of links that could possibly exist between them. Betweenness centrality reflects the role played by a node (in our case, a gene) in the global network architecture and, for the yeast INGI, is defined as the fraction of shortest paths between *g*_*i *_and *g*_*j *_passing through *g*. We computed the betweenness centrality based on shortest paths via all types of interaction (*inbet*) as well as based on shortest paths via each type of interaction (*inbetppi*, *inbetmet *and *inbetreg*). Closeness centrality (*cent*) measures how close a node (in our case, a gene) is to all others in the network and, for the yeast INGI, is defined as the mean shortest path between *g *and all other genes reachable from it. Identicalness is the number of genes with identical network topological characteristics. All these network topological features, except for the betweenness centrality-related features, were calculated by a program written in a Mathematica^® ^6.0 notebook. The betweenness centrality-related features were calculated by the Python package *NetworkX *[54].

#### Cellular localization and biological process annotation of yeast genes

We determined the cellular component in which a yeast gene product acts and the biological process in which a yeast gene is involved by using the Saccharomyces Genome Database (SGD) Gene Ontology (GO) Slim Mapper [55]. The SGD GO-Slim Mapper maps annotations of a group of genes to more general GO terms. Among GO Slim sets available at SGD, we selected cellular component and biological process terms from the Super GO-Slim set, a collection of high-level GO terms. For cellular localization annotation, genes annotated to terms rather than "cytoplasm", "endoplasmic reticulum", "mitochondrion" and "nucleus" were reannotated to one of these terms or to a new term named "other localization". For biological process annotation, genes annotated to terms rather than "cell cycle", "metabolic process", "signal transduction", "transcription" and "transport" were reannotated to one of these terms or to a new term named "other process".

### Classifier design, training and evaluation

#### Construction of datasets for classifier training and evaluation

We defined "essential genes" as those genes whose deletion leads to an inviable yeast organism cultured on rich glucose medium. We obtained the dataset containing the classification of yeast genes in essential or non-essential from Giaever *et al*. [4]. After downloading the dataset, we removed from it genes classified as dubious in SGD and converted the name of remaining genes to their systematic names as provided by the SGD Nomenclature Conventions [23].

As this dataset of classified genes is an imbalanced dataset, i.e., the number of non-essential genes is much larger than the number of essential genes, and it has been known that data imbalance degrades the performance of machine learning algorithms [17], we built balanced datasets from the original imbalanced dataset by using an undersampling scheme as follows: (1) first, we split the dataset of classified genes into two subsets: "essential genes set", containing 1,024 essential gene entries, and "non-essential genes set", containing 4,097 non-essential gene entries; (2) second, we selected all entries from the essential genes set (1,024 entries) and randomly selected 1,024 entries from the non-essential genes set; (3) we then created the balanced dataset containing the 2,048 selected entries with random distribution of the essential gene and non-essential gene entries. This procedure was repeated 10 times in order to generate 10 different balanced datasets containing different sets of non-essential gene entries.

To compare the predictability of essential genes by individual training features with that of different groups of training features, we generated, from the balanced datasets, different subsets containing different combinations of training features as detailed in Additional file 2.

#### Classifier design

We used WEKA (Waikato Environment for Knowledge Analysis) software package, a collection of machine learning algorithms for data mining tasks [56], for designing, training and evaluating the classifiers applied to prediction of essential genes. Among classifiers that we evaluated, the one that provided the best performance was an ensemble of eight decision tree algorithms using the meta-classifier "Vote", a WEKA's implementation of the voting algorithm that combines the output predictions of each classifier by different rules [57]. We combined the classifiers by the average rule, where the output predictions computed by the individual classifiers for each class are averaged and this average is used in its decision [57]. The classifiers composing our model were: (1) REPtree [56], (2) naive bayes tree [58], (3) random tree [56], (4) random forest [59], (5) J48, a WEKA's implementation of the C4.5 decision tree [46], with minimum number of 32 instances per leaf, (6) best-first decision tree with minimum number of 32 instances at the terminal nodes [60], (7) logistic model tree [61] and (8) alternating decision tree with 25 boost iterations [62]. In addition, we applied the bootstrap aggregating (bagging) approach [63] to each classifier. Parameters values for each classifier are provided in the Additional file 6.

#### Classifier training and evaluation

For each of the 10 balanced datasets, we trained our classifier on half of entries and the other half was used to evaluate the classifier performance, totaling 10 runs of training and evaluation. For these runs, we generated a receiver operating characteristic (ROC) curve and calculated the area under the ROC curve (AUC). The ROC curve is a plot of the true positive rate versus false positive rate and indicates the probability of a true positive prediction as a function of the probability of a false positive prediction for all possible threshold values [64]. AUC is a widely used summary measure of the ROC curve and is equivalent to the probability that a randomly chosen negative example (in our case, a non-essential gene) will have a smaller estimated probability of belonging to the positive class than a randomly chosen positive example (in our case, an essential gene) [65].

We used the web server version of the StAR (**St**atistical **A**nalysis of **R**OC curves) software [24] for calculating the true and false positive rates and the AUC values and for generating the ROC curves. The statistical comparison of AUC values derived from the different datasets was also performed by StAR by means of a nonparametric statistical method based on the Mann-Whitney U-statistic for comparing distributions of values from two samples [18] with a significance level (*P*) of 0.01.

### Determination of rules for gene essentiality

The determination of rules for gene essentiality was performed by analyzing decision trees generated through the training of J48 algorithm on balanced datasets containing all training data. We used two different values of the parameter "number of objects per leaf" of J48 algorithm for generating two different types of decision trees: 64 for more detailed trees and 128 for more simplified trees [56]. For each balanced dataset, then, we obtained two decision trees (detailed and simplified) and manually inspected all the 20 generated decision trees for determining the general rules for gene essentiality. The remaining parameters values for producing decision trees by J48 algorithm training are provided in the Additional file 6 and all decision trees are provided in text format in the Additional file 5.

## Authors' contributions

MLA obtained all interaction data, constructed the network, designed and analyzed the classifier performance, pursued the biological interpretation of results and drafted the manuscript. NL conceived, designed and directed the project and implemented the program for calculation of network topological features. All authors read and approved the final manuscript.

## Supplementary Material

Additional file 1 **Network topological features**. This file includes a table showing the functions and descriptions of the twelve network topological features used as learning attributes for training the classifier algorithm Click here for file

Additional file 2 **Statistical pairwise comparison of predictors**. This file includes tables showing the pairwise comparison of predictors with the p-values of AUC differences between each pair of predictors. Click here for file

Additional file 3 **ROC curves and AUC values demonstrating the effect of removal of individual or small sets of network topological features**. File containing ROC curves for classifiers trained on datasets whose learning attributes were different sets of network topological features in which each set lacks one of the topological features or a small group of 2-4 topological features. Click here for file

Additional file 4 **List of the 470 yeast genes predicted to be non-essential**. Tab-limited text file containing the 470 genes classified as non-essential with their essentiality scores, actual essentiality statuses and, if applicable, the Pubmed references showing their essentiality statuses. Click here for file

Additional file 5 **J48 decision trees**. This file contains all 10 decision trees generated by training the J48 algorithm on the 10 balanced datasets with all available data as learning attributes. Decision trees are represented in text format (raw output generated by WEKA). Click here for file

Additional file 6 **Parameters used to train the meta-classifier and J48**. File containing all parameters values used to train the meta-classifier for essential gene prediction and all parameters values used to train the J48 algorithm to generate decision trees for discovery of cellular rules for essentiality. Click here for file
