# Supplementary material for: Towards the prediction of essential genes by integration of network topology, cellular localization and biological process information
Source: BMC Bioinformatics. 2009 Sep 16;10:290. doi: 10.1186/1471-2105-10-290 (PMC2753850; doi:10.1186/1471-2105-10-290)
Supplement: Additional file 1 — Network topological features. This file includes a table showing the functions and descriptions of the twelve network topological features used as learning attributes for training the classifier algorithm [file 1471-2105-10-290-S1.PDF]

Functions and descriptions of the twelve network topological features used as learning attributes for training the classifier algorithm

Feature	Function	Description
Degree centrality related to protein physical interaction	ppi	Number of undirected links to gene g representing the number of protein physical interactions.
Indegree related to metabolic interaction	$metin$	Number of incoming links to gene g representing the number of reactants participating in a metabolic reaction catalyzed by the enzyme encoded by g
Outdegree related to metabolic interaction	$metout$	Number of outgoing links from gene g representing the number of products generated by the metabolic reaction catalyzed by the enzyme encoded by g
Indegree related to transcriptional regulation interaction	$regin$	Number of incoming links to gene g representing the number of transcription factors regulating g
Outdegree related to transcriptional regulation interaction	$regout$	Number of outgoing links from gene g representing the number of genes regulated by the transcription factor encoded by g
Clustering coefficient	$c = \frac{2n_g}{k_g(k_g - 1)}$	n_g is the number of links connecting the neighbors of g and k_g is the number of links connecting g to its neighbors.
Betweenness centrality related to all interactions	$inbet(g) = \sum_{g_i \neq g \neq g_j} \frac{\sigma_{g_i g_j}(g)}{\sigma_{g_i g_j}}$	$\sigma_{g_i g_j}$ is the number of shortest paths between g_i and g_j and $\sigma_{g_i g_j}(g)$ is the number of shortest paths between g_i and g_j passing through g. Shortest paths via all types of interactions.
Betweenness centrality related to protein physical interactions	$inbetppi(g) = \sum_{g_i \neq g \neq g_j} \frac{\sigma_{g_i g_j}(g)}{\sigma_{g_i g_j}}$	$\sigma_{g_i g_j}$ is the number of shortest paths between g_i and g_j and $\sigma_{g_i g_j}(g)$ is the number of shortest paths between g_i and g_j passing through g. Shortest paths via protein physical of interactions
Betweenness centrality related to metabolic interactions	$inbetmet(g) = \sum_{g_i \neq g \neq g_j} \frac{\sigma_{g_i g_j}(g)}{\sigma_{g_i g_j}}$	$\sigma_{g_i g_j}$ is the number of shortest paths between g_i and g_j and $\sigma_{g_i g_j}(g)$ is the number of shortest paths between g_i and g_j passing through g. Shortest paths via metabolic interactions.
Betweenness centrality related to transcriptional regulation interactions	$inbetreg(g) = \sum_{g_i \neq g \neq g_j} \frac{\sigma_{g_i g_j}(g)}{\sigma_{g_i g_j}}$	$\sigma_{g_i g_j}$ is the number of shortest paths between g_i and g_j and $\sigma_{g_i g_j}(g)$ is the number of shortest paths between g_i and g_j passing through g. Shortest paths via transcriptional regulation interactions.
Closeness centrality	$cent = \frac{n}{\sum_{g_j} d(g, g_j)}$	$d(g, g_j)$ is the shortest distance via all types of interactions between genes g and g_j; n is the number of genes in the network
Identicalness	$ident$	Number of genes with identical network topological characteristics
