# Supplementary material for: Towards the prediction of essential genes by integration of network topology, cellular localization and biological process information
Source: BMC Bioinformatics. 2009 Sep 16;10:290. doi: 10.1186/1471-2105-10-290 (PMC2753850; doi:10.1186/1471-2105-10-290)
Supplement: Additional file 3 — ROC curves and AUC values demonstrating the effect of removal of individual or small sets of network topological features. File containing ROC curves for classifiers trained on datasets whose learning attributes were different sets of network topological features in which each set lacks one of the topological features or a small group of 2-4 topological features. [file 1471-2105-10-290-S3.PDF]

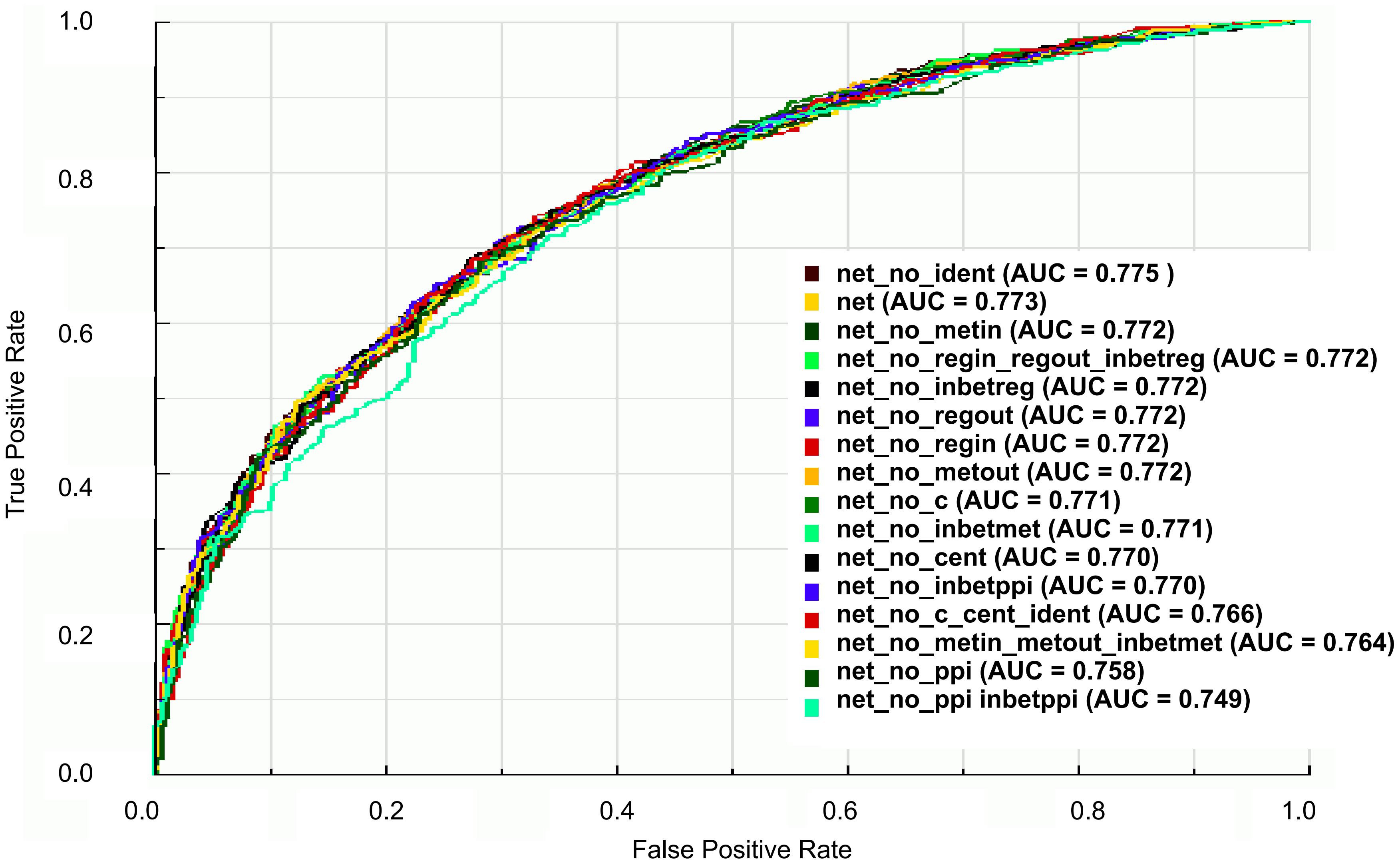

1.0
0.8
0.6
0.4
0.2
0.0
True Positive Rate
0.0
0.2
0.4
0.6
0.8
1.0
False Positive Rate
net_no_ident (AUC = 0.775)
net (AUC = 0.773)
net_no_metin (AUC = 0.772)
net_no_regin_regout_inbetreg (AUC = 0.772)
net_no_inbetreg (AUC = 0.772)
net_no_regout (AUC = 0.772)
net_no_regin (AUC = 0.772)
net_no_metout (AUC = 0.772)
net_no_c (AUC = 0.771)
net_no_inbetmet (AUC = 0.771)
net_no_cent (AUC = 0.770)
net_no_inbetppi (AUC = 0.770)
net_no_c_cent_ident (AUC = 0.766)
net_no_metin_metout_inbetmet (AUC = 0.764)
net_no_ppi (AUC = 0.758)
net_no_ppi inbetppi (AUC = 0.749)

ROC curves and AUC values for the classifiers trained on balanced datasets whose learning attributes are all network topological features or sets of network topological features from which one or a small group of the features was removed.

These ROC curves were obtained by plotting the true positive rates versus the false positive rates for essential gene prediction calculated from the probability predictions generated by training our classifier on the balanced dataset 9 (see Figure 1 in paper) whose learning attributes were all of the network topological features or sets of network topological features from which one or a small group of the features was removed. The classifier training was performed by the WEKA software package and the calculations of true and false positives rates and AUC values were performed by the server version of the StAR software. "net" is the dataset with all network topological features as learning attributes; “net_no_features”, in which “features” can be "ppi", "inbetppi", "inbet", "c", "cent", "regin", "metin", "regout". "metout", "inbetmet", "ident", "inbetreg" or the combinations of some features, are datasets with all network topological features except one or the combinations of the following network topological features as learning attribute: number of protein physical interactions (ppi), betweenness centrality for the protein physical interactions (inbetppi), betweenness centrality for all types of interactions (inbet), clustering coefficient (c), closeness centrality (cent), number of regulating transcription factor (regin), number of reactants participating in a metabolic reaction catalyzed by the enzyme encoded by the gene (metin), number of genes regulated by the transcription factor encoded by the gene (regout), number of products generated in a metabolic reaction catalyzed by the enzyme encoded by the gene (metout), betweenness centrality for the metabolic interactions (inbetmet), number of genes with identical topological features (ident) and betweenness centrality for the transcriptional regulation interactions (inbetreg). For more details on network topological features, see Additional File 1.
