# Supplementary material for: Towards the prediction of essential genes by integration of network topology, cellular localization and biological process information
Source: BMC Bioinformatics. 2009 Sep 16;10:290. doi: 10.1186/1471-2105-10-290 (PMC2753850; doi:10.1186/1471-2105-10-290)
Supplement: Additional file 5 — J48 decision trees. This file contains all 10 decision trees generated by training the J48 algorithm on the 10 balanced datasets with all available data as learning attributes. Decision trees are represented in text format (raw output generated by WEKA). [file 1471-2105-10-290-S5.PDF]

J48 algorithm-generated Decision Trees in Text Format

1. Decision Trees generated with 128 instances per leaf (simplified decision trees):

Decision tree of Balanced Dataset 1:

```
ppi <= 8
|   metabolicprocess = true
|   |   regin <= 3
|   |   |   nucleus = true: essential (153.0/59.0)
|   |   |   nucleus = false: nonessential (215.0/84.0)
|   |   regin > 3: nonessential (158.0/42.0)
|   metabolicprocess = false: nonessential (445.0/69.0)
ppi > 8
|   nucleus = true: essential (590.0/118.0)
|   nucleus = false
|   |   regin <= 3
|   |   |   ppi <= 18: nonessential (146.0/72.0)
|   |   |   ppi > 18: essential (172.0/57.0)
|   |   regin > 3: nonessential (169.0/76.0)
```

Decision tree of Balanced Dataset 2:

```
ppi <= 6
|   metabolicprocess = false: nonessential (385.0/51.0)
|   metabolicprocess = true
|   |   nucleus = false: nonessential (289.0/88.0)
|   |   nucleus = true: essential (173.0/83.0)
ppi > 6
|   nucleus = false
|   |   ppi <= 13: nonessential (224.0/98.0)
|   |   ppi > 13: essential (332.0/134.0)
|   nucleus = true: essential (645.0/146.0)
```

Decision tree of Balanced Dataset 3:

```
ppi <= 12
|   metabolicprocess = false: nonessential (530.0/102.0)
|   metabolicprocess = true
|   |   nucleus = false: nonessential (374.0/137.0)
|   |   nucleus = true
|   |   |   c <= 0.137681: nonessential (128.0/57.0)
|   |   |   c > 0.137681: essential (151.0/55.0)
ppi > 12: essential (865.0/233.0)
```

Decision tree of Balanced Dataset 4:

```
ppi <= 7
|   metabolicprocess = true
|   |   regin <= 3
|   |   |   nucleus = false: nonessential (190.0/76.0)
|   |   |   nucleus = true: essential (149.0/63.0)
|   |   regin > 3: nonessential (148.0/37.0)
|   metabolicprocess = false: nonessential (455.0/60.0)
ppi > 7
|   nucleus = false
|   |   transport = false
|   |   |   inbet <= 0.000233: nonessential (188.0/89.0)
```

```
|   |   |   inbet > 0.000233: essential (145.0/61.0)
|   |   transport = true: essential (150.0/44.0)
|   nucleus = true: essential (623.0/137.0)
```

Decision tree of Balanced Dataset 5:

```
ppi <= 6: nonessential (848.0/229.0)
ppi > 6
|   nucleus = true: essential (650.0/151.0)
|   nucleus = false
|   |   transport = false
|   |   |   inbet <= 0.000289: nonessential (242.0/106.0)
|   |   |   inbet > 0.000289: essential (130.0/49.0)
|   |   transport = true: essential (178.0/69.0)
```

Decision tree of Balanced Dataset 6:

```
ppi <= 6
|   metabolicprocess = false: nonessential (402.0/51.0)
|   metabolicprocess = true
|   |   regout <= 3
|   |   |   ppi <= 2: nonessential (159.0/69.0)
|   |   |   ppi > 2: essential (144.0/68.0)
|   |   regout > 3: nonessential (134.0/33.0)
ppi > 6
|   nucleus = true: essential (646.0/147.0)
|   nucleus = false
|   |   metabolicprocess = false: nonessential (249.0/117.0)
|   |   metabolicprocess = true: essential (314.0/135.0)
```

Decision tree of Balanced Dataset 7:

```
ppi <= 6
|   metabolicprocess = true
|   |   nucleus = true: essential (162.0/72.0)
|   |   nucleus = false: nonessential (283.0/88.0)
|   metabolicprocess = false: nonessential (382.0/51.0)
ppi > 6
|   nucleus = true: essential (668.0/169.0)
|   nucleus = false
|   |   regin <= 3
|   |   |   c <= 0.160594: nonessential (189.0/93.0)
|   |   |   c > 0.160594: essential (175.0/56.0)
|   |   regin > 3: nonessential (189.0/84.0)
```

Decision tree of Balanced Dataset 8:

```
ppi <= 6
|   metabolicprocess = true
|   |   nucleus = false: nonessential (283.0/88.0)
|   |   nucleus = true: essential (157.0/67.0)
|   metabolicprocess = false: nonessential (422.0/51.0)
ppi > 6
|   nucleus = false
|   |   regin <= 3: essential (362.0/150.0)
|   |   regin > 3: nonessential (187.0/84.0)
|   nucleus = true: essential (637.0/138.0)
```

Decision tree of Balanced Dataset 9:

```
ppi <= 7
|   metabolicprocess = true
|   |   regin <= 3
|   |   |   nucleus = true: essential (136.0/50.0)
|   |   |   nucleus = false: nonessential (189.0/76.0)
|   |   regin > 3: nonessential (142.0/37.0)
|   metabolicprocess = false: nonessential (443.0/60.0)
ppi > 7
|   nucleus = true: essential (624.0/138.0)
|   nucleus = false
|   |   inbetppi <= 0.000966
|   |   |   regin <= 3: essential (250.0/114.0)
|   |   |   regin > 3: nonessential (130.0/54.0)
|   |   inbetppi > 0.000966: essential (134.0/45.0)
```

Decision tree of Balanced Dataset 10:

```
ppi <= 6
|   metabolicprocess = true
|   |   nucleus = false: nonessential (271.0/88.0)
|   |   nucleus = true: essential (160.0/70.0)
|   metabolicprocess = false: nonessential (403.0/51.0)
ppi > 6
|   nucleus = false
|   |   ppi <= 18
|   |   |   c <= 0.155556: nonessential (151.0/51.0)
|   |   |   c > 0.155556: essential (160.0/73.0)
|   |   ppi > 18: essential (262.0/104.0)
|   nucleus = true: essential (641.0/142.0)
```

2. Decision Trees generated with 64 instances per leaf (detailed decision trees):

Decision tree of Balanced Dataset 1:

```
ppi <= 8
|   metabolicprocess = true
|   |   regin <= 3
|   |   |   nucleus = true: essential (153.0/59.0)
|   |   |   nucleus = false
|   |   |   |   metin <= 0: nonessential (147.0/46.0)
|   |   |   |   metin > 0: essential (68.0/30.0)
|   |   regin > 3: nonessential (158.0/42.0)
|   metabolicprocess = false: nonessential (445.0/69.0)
ppi > 8
|   nucleus = true: essential (590.0/118.0)
|   nucleus = false
|   |   transport = false
|   |   |   metabolicprocess = true
|   |   |   |   inbetppi <= 0.000616: nonessential (136.0/63.0)
|   |   |   |   inbetppi > 0.000616: essential (108.0/36.0)
|   |   |   metabolicprocess = false: nonessential (77.0/27.0)
|   |   transport = true
|   |   |   regin <= 2: essential (97.0/30.0)
|   |   |   regin > 2: nonessential (69.0/34.0)
```

Decision tree of Balanced Dataset 2:

```
ppi <= 6
|   metabolicprocess = false: nonessential (385.0/51.0)
|   metabolicprocess = true
|   |   nucleus = false: nonessential (289.0/88.0)
|   |   nucleus = true
|   |   |   regin <= 1: essential (73.0/29.0)
|   |   |   regin > 1: nonessential (100.0/46.0)
ppi > 6
|   nucleus = false
|   |   cytoplasm = true
|   |   |   er = false
|   |   |   |   regin <= 2: essential (212.0/86.0)
|   |   |   |   regin > 2
|   |   |   |   |   ppi <= 13: nonessential (69.0/25.0)
|   |   |   |   |   ppi > 13: essential (112.0/51.0)
|   |   |   er = true: essential (78.0/30.0)
|   |   cytoplasm = false: nonessential (85.0/36.0)
|   nucleus = true: essential (645.0/146.0)
```

Decision tree of Balanced Dataset 3:

```
ppi <= 12
|   metabolicprocess = false
|   |   ppi <= 5: nonessential (379.0/44.0)
|   |   ppi > 5
|   |   |   transport = true: essential (65.0/31.0)
|   |   |   transport = false: nonessential (86.0/24.0)
|   metabolicprocess = true
|   |   inbetreg <= 0.000149
|   |   |   nucleus = false
|   |   |   |   inbetppi <= 0.00014: nonessential (271.0/91.0)
|   |   |   |   inbetppi > 0.00014: essential (66.0/29.0)
|   |   |   nucleus = true: essential (227.0/83.0)
|   |   inbetreg > 0.000149: nonessential (89.0/18.0)
ppi > 12
|   nucleus = false
|   |   regin <= 3: essential (240.0/89.0)
|   |   regin > 3: nonessential (122.0/60.0)
|   nucleus = true: essential (503.0/82.0)
```

Decision tree of Balanced Dataset 4:

```
ppi <= 7
|   metabolicprocess = true
|   |   nucleus = false
|   |   |   regin <= 3
|   |   |   |   metin <= 0: nonessential (126.0/42.0)
|   |   |   |   metin > 0: essential (64.0/30.0)
|   |   |   regin > 3: nonessential (113.0/23.0)
|   |   nucleus = true
|   |   |   c <= 0.084211: nonessential (71.0/30.0)
|   |   |   c > 0.084211: essential (113.0/43.0)
|   metabolicprocess = false: nonessential (455.0/60.0)
ppi > 7
|   nucleus = false
|   |   transport = false
|   |   |   metabolicprocess = true
|   |   |   |   inbet <= 0.000282
|   |   |   |   |   inbetppi <= 0.000291: nonessential (74.0/33.0)
```

```
|   |   |   |   |   inbetppi > 0.000291: essential (69.0/27.0)
|   |   |   |   inbet > 0.000282: essential (103.0/33.0)
|   |   |   metabolicprocess = false: nonessential (87.0/28.0)
|   |   transport = true: essential (150.0/44.0)
|   nucleus = true: essential (623.0/137.0)
```

Decision tree of Balanced Dataset 5:

```
ppi <= 6
|   metabolicprocess = true
|   |   regin <= 4
|   |   |   nucleus = true: essential (140.0/57.0)
|   |   |   nucleus = false: nonessential (207.0/75.0)
|   |   regin > 4: nonessential (104.0/20.0)
|   metabolicprocess = false: nonessential (397.0/51.0)
ppi > 6
|   nucleus = true: essential (650.0/151.0)
|   nucleus = false
|   |   er = false
|   |   |   transport = false
|   |   |   |   metabolicprocess = true
|   |   |   |   |   inbetppi <= 0.000696: nonessential (150.0/70.0)
|   |   |   |   |   inbetppi > 0.000696: essential (86.0/26.0)
|   |   |   |   metabolicprocess = false: nonessential (97.0/32.0)
|   |   |   transport = true
|   |   |   |   c <= 0.138258: nonessential (67.0/33.0)
|   |   |   |   c > 0.138258: essential (77.0/24.0)
|   |   er = true: essential (73.0/25.0)
```

Decision tree of Balanced Dataset 6:

```
ppi <= 6
|   metabolicprocess = false: nonessential (402.0/51.0)
|   metabolicprocess = true
|   |   regout <= 3
|   |   |   nucleus = true: essential (125.0/48.0)
|   |   |   nucleus = false: nonessential (178.0/68.0)
|   |   regout > 3: nonessential (134.0/33.0)
ppi > 6
|   nucleus = true
|   |   ppi <= 30
|   |   |   c <= 0.083333: nonessential (69.0/30.0)
|   |   |   c > 0.083333: essential (325.0/87.0)
|   |   ppi > 30: essential (252.0/21.0)
|   nucleus = false
|   |   metin <= 0
|   |   |   ppi <= 18
|   |   |   |   transport = false: nonessential (147.0/50.0)
|   |   |   |   transport = true: essential (102.0/50.0)
|   |   |   ppi > 18
|   |   |   |   regout <= 2: essential (140.0/46.0)
|   |   |   |   regout > 2: nonessential (101.0/50.0)
|   |   metin > 0: essential (73.0/23.0)
```

Decision tree of Balanced Dataset 7:

```
  metabolicprocess = true
|   |   nucleus = true: essential (162.0/72.0)
|   |   nucleus = false: nonessential (283.0/88.0)
```

```
|   metabolicprocess = false: nonessential (382.0/51.0)
ppi > 6
|   nucleus = true
|   |   ppi <= 22
|   |   |   c <= 0.088889: nonessential (64.0/25.0)
|   |   |   c > 0.088889: essential (245.0/79.0)
|   |   ppi > 22: essential (359.0/51.0)
|   nucleus = false
|   |   er = false
|   |   |   regin <= 3
|   |   |   |   metabolicprocess = true
|   |   |   |   |   inbetppi <= 0.000656: nonessential (97.0/47.0)
|   |   |   |   |   inbetppi > 0.000656: essential (69.0/19.0)
|   |   |   |   metabolicprocess = false
|   |   |   |   |   c <= 0.153846: nonessential (84.0/37.0)
|   |   |   |   |   c > 0.153846: essential (64.0/21.0)
|   |   |   regin > 3: nonessential (172.0/71.0)
|   |   er = true: essential (67.0/19.0)
```

Decision tree of Balanced Dataset 8:

```
ppi <= 6
|   metabolicprocess = true
|   |   nucleus = false: nonessential (283.0/88.0)
|   |   nucleus = true
|   |   |   regin <= 1: essential (64.0/20.0)
|   |   |   regin > 1: nonessential (93.0/46.0)
|   metabolicprocess = false: nonessential (422.0/51.0)
ppi > 6
|   nucleus = false
|   |   er = false
|   |   |   ppi <= 22
|   |   |   |   mitochondrion = false: nonessential (226.0/97.0)
|   |   |   |   mitochondrion = true: essential (70.0/34.0)
|   |   |   ppi > 22: essential (184.0/69.0)
|   |   er = true: essential (69.0/21.0)
|   nucleus = true: essential (637.0/138.0)
```

Decision tree of Balanced Dataset 9:

```
ppi <= 7
|   metabolicprocess = true
|   |   regin <= 3
|   |   |   nucleus = true: essential (136.0/50.0)
|   |   |   nucleus = false: nonessential (189.0/76.0)
|   |   regin > 3: nonessential (142.0/37.0)
|   metabolicprocess = false: nonessential (443.0/60.0)
ppi > 7
|   nucleus = true: essential (624.0/138.0)
|   nucleus = false
|   |   er = false
|   |   |   inbetppi <= 0.001192
|   |   |   |   inbet <= 0.000328
|   |   |   |   |   transport = false: nonessential (182.0/82.0)
|   |   |   |   |   transport = true: essential (108.0/46.0)
|   |   |   |   inbet > 0.000328: nonessential (70.0/28.0)
|   |   |   inbetppi > 0.001192: essential (88.0/27.0)
|   |   er = true: essential (66.0/20.0)
```

Decision tree of Balanced Dataset 10

```
ppi <= 6
|   metabolicprocess = true
|   |   nucleus = false: nonessential (271.0/88.0)
|   |   nucleus = true: essential (160.0/70.0)
|   metabolicprocess = false: nonessential (403.0/51.0)
ppi > 6
|   nucleus = false
|   |   metabolicprocess = true
|   |   |   inbet <= 0.000425
|   |   |   |   regin <= 3
|   |   |   |   |   ppi <= 18: nonessential (102.0/50.0)
|   |   |   |   |   ppi > 18: essential (75.0/26.0)
|   |   |   |   regin > 3: nonessential (67.0/30.0)
|   |   |   inbet > 0.000425: essential (71.0/21.0)
|   |   metabolicprocess = false
|   |   |   transport = false: nonessential (99.0/32.0)
|   |   |   transport = true
|   |   |   |   c <= 0.179402: nonessential (85.0/37.0)
|   |   |   |   c > 0.179402: essential (74.0/26.0)
|   nucleus = true: essential (641.0/142.0)
```

3. Decision Tree generated by training the J48 algorithm on balanced dataset 8 with all features plus nucleolus, nucleoplasm, nuclear chromosome and nuclear envelope (with 128 instances per leaf):

```
nucleolus = false
|   ppi <= 6: nonessential (859.0/225.0)
|   ppi > 6
|   |   ppi <= 18
|   |   |   regin <= 3: essential (339.0/146.0)
|   |   |   regin > 3: nonessential (147.0/69.0)
|   |   ppi > 18: essential (607.0/162.0)
nucleolus = true: essential (159.0/11.0)
```
