# Supplementary material for: Towards the prediction of essential genes by integration of network topology, cellular localization and biological process information
Source: BMC Bioinformatics. 2009 Sep 16;10:290. doi: 10.1186/1471-2105-10-290 (PMC2753850; doi:10.1186/1471-2105-10-290)
Supplement: Additional file 6 — Parameters used to train the meta-classifier and J48. File containing all parameters values used to train the meta-classifier for essential gene prediction and all parameters values used to train the J48 algorithm to generate decision trees for discovery of cellular rules for essentiality. [file 1471-2105-10-290-S6.PDF]

A. Parameters (**) of the decision tree-based meta-classifier used to predict essential genes (based on the graphic user interface of WEKA software package):

Meta-classifier: Vote
**classifiers: 8 weka.classifiers.Classifier (decision tree classifiers with bootstrap aggregating [bagging] approach as described below)
**combinationRule: Average of Probabilities
**debug: False
**seed: 1

-- We applied the class "weka.classifiers.meta.Bagging" (bootstrap aggregating [bagging]) approach to each classifier (see details in the section "Methods" in paper) with the following parameters:
**bagSizePercent: 100
**calcOutOfBag: false
**classifier: one of the 8 decision tree classifiers whose parameters are described below.
**debug: False
**numiterations: 20
**seed: 1

Parameters of the 8 bagged decision tree classifiers:

1. REPTree
**debug: False
** maxDepth: -1
** minNum: 2.0
**minVarianceProp: 0.0010
**noPrunning: False
**numFolds: 3
**seed: 1

2. Naive bayes tree (NBtree)
*debug: False

3. Random Tree
**KValue: 1
**debug: false
**maxDepth: 0
**minNum: 1.0
**seed: 1

4. Random Forest
**debug: False
**maxDepth: 0
**numFeatures: 0
**numTrees: 10
seed: 1

5. J48
**binarySplits: False
** confidenceFactor: 0.5
**debug: False
**minNumObj: 32
**numFolds: 3
**reducedErrorPruning: False
**saveInstanceData: False
**seed: 1
**unpruned : False
**useLaplace: False

6. Best-first decision tree (BFtree)
**debug: False
**heuristic: True

**minNumObj: 32
**numFoldsPruning: 5
**pruningStrategy: Post-pruning
**seed: 1
**sizePer: 1.0
**useErrorRate: True
**useGini: True
**useOneSE: False

7. Logistic model tree (LMT)
**convertNominal: False
**debug: False
**errorOnProbabilities: False
**fastRegression: True
**minNumInstances: 15
**numBoostingIterations: -1
**splitOnResiduals: False
**useAIC: False
**weightTrimBeta: 0.0

8. Alternating decision tree (ADtree)
**debug: False
**numOfBoostingIterations: 25
**randomSeed: 0
**saveInstanceData: False
**searchPath: Expand the heaviest path

B. Parameters (**) of the J48 classifier used to generated the decision trees for determination of cellular rules for gene essentiality (based on the graphic user interface of WEKA software package):

For simplified decision trees (see Additional File 3 and "Methods" in paper):

**binarySplits: False
** confidenceFactor: 0.5
**debug: False
****minNumObj: 128**
**numFolds: 3
**reducedErrorPruning: False
**saveInstanceData: False
**seed: 1
**unpruned : False
**useLaplace: False

For detailed decision trees (see Additional File 3 and "Methods" in paper):

**binarySplits: False
** confidenceFactor: 0.5
**debug: False
****minNumObj: 64**
**numFolds: 3
**reducedErrorPruning: False
**saveInstanceData: False
**seed: 1
**unpruned : False
**useLaplace: False
